# Psychotic Spectrum Symptoms in Adults with Autism Spectrum Disorder and in Their First-Degree Relatives

**DOI:** 10.3390/brainsci16030307

**Published:** 2026-03-13

**Authors:** Benedetta Nardi, Francesca Parri, Stefano Pini, Federico Giovannoni, Cristiana Pronestì, Silvia Tarantino, Gabriele Massimetti, Ivan Mirko Cremone, Liliana Dell’Osso, Barbara Carpita

**Affiliations:** Department of Clinical and Experimental Medicine, Section of Psychiatry, University of Pisa, 67 Via Roma, 56126 Pisa, Italy; benedetta.nardi@live.it (B.N.); stefano.pini@unipi.it (S.P.); f.giovannoni1@studenti.unipi.it (F.G.); c.pronesti@studenti.unipi.it (C.P.); s.tarantino2@studenti.unipi.it (S.T.); gabriele.massimetti@unipi.it (G.M.); ivan.cremone@gmail.com (I.M.C.); liliana.dellosso@gmail.com (L.D.); barbara.carpita1986@gmail.com (B.C.)

**Keywords:** autism spectrum disorder, autistic traits, broad autism phenotype, psychosis, psychotic spectrum

## Abstract

**Objectives**: Autism Spectrum Disorder (ASD) and psychotic disorders have long been considered separate diagnostic entities, yet increasing evidence highlights shared neurodevelopmental mechanisms and symptom overlap. Psychotic-like experiences have been frequently reported in individuals with ASD, while subthreshold autistic traits (ATs) in first-degree relatives may also confer vulnerability to psychotic symptoms. This cross-sectional study aimed to compare psychotic spectrum manifestations among adults with ASD, their first-degree relatives (BAP), and controls (HCs), to explore associations between psychotic and ATs, and to evaluate whether psychotic symptoms predict diagnostic group membership. **Methods**: 22 adults with ASD, 22 BAP, and 24 HCs were evaluated with the Psychotic Spectrum–Self Report (PSY-SR) and the Adult Autism Subthreshold Spectrum (AdAS Spectrum). **Results**: ASD participants scored significantly higher on the PSY-SR. BAP individuals showed higher PSY-SR total scores compared to HCs, though less severe than in ASD. All PSY-SR domains positively correlated with all AdAS Spectrum domains, with few exceptions. Multinomial regressions showed that higher PSY-SR total scores significantly predicted ASD and BAP membership, and that the PSY-SR *Paranoid* domain score specifically predicted inclusion in both groups in relation to HCs. **Conclusions**: Psychotic spectrum symptoms are elevated not only in individuals with ASD but also among first-degree relatives, supporting a continuum linking autistic and psychotic vulnerabilities. The strong association between paranoid symptoms and ATs highlights a dimension of potential clinical relevance for early identification and assessment. These findings reinforce shared neurodevelopmental pathways between the autism and psychosis spectra and underscore the importance of dimensional approaches across diagnostic categories.

## 1. Introduction

Autism Spectrum Disorder (ASD) is a complex neurodevelopmental condition characterized by persistent difficulties in social communication and interaction, alongside restricted, repetitive patterns of behavior, interests, or activities [1]. Beyond these core features, individuals with ASD often present with heterogeneous profiles, including atypical cognitive functioning, significant executive deficits, and difficulties in processing and integrating sensory and social information [2]. The clinical manifestations of ASD can vary widely in both severity and presentation, ranging from individuals with profound impairments, including intellectual disability and absence of verbal communication, to those with high-functioning profiles, formerly referred to as Asperger’s Syndrome, who may maintain adaptive functioning in work and daily life despite marked social and relational difficulties [3,4].

While ASD is recognized as a distinct diagnostic category, there is a growing body of literature highlighting overlaps with other psychiatric conditions, particularly those within the psychotic spectrum. Historically, autism and schizophrenia were closely linked in early psychiatric literature. Notably, Bleuler included “autism” among the “four A’s” of schizophrenia (along with affective blunting, alogia, and ambivalence), conceptualizing it as a core feature of what he termed “schizophrenia” [5,6]. The formal diagnostic separation of autism and schizophrenia emerged with the third edition of the *Diagnostic and Statistical Manual for Mental Disorders* (DSM-III) [7], which established mutual exclusivity criteria. However, advances in neurobiology and genetics have since challenged the strict dichotomy between the two conditions. Recent evidence suggests that ASD and psychosis may share common neurodevelopmental pathways. Studies have pointed out converging alterations in molecular mechanisms involved in early brain development, including synaptic pruning, gene expression regulation, and neural plasticity [8,9,10,11]. This has led to increasing support for a dimensional view of psychopathology, with overlapping spectra rather than rigid diagnostic boundaries [12]. Moreover, clinically, individuals with ASD may exhibit psychotic-like experiences such as paranoid ideation, perceptual distortions, or dissociative symptoms, even in the absence of a formal psychotic disorder. Indeed, a recent meta-analysis has reported how psychotic experiences are significantly more prevalent in subjects with ASD compared to the general population [13]. Conversely, patients with psychosis, schizophrenia, or schizotypal traits frequently show impairments resembling autistic features, including social withdrawal, communicative challenges, and cognitive rigidity [8,14,15,16]. These findings have reinforced the notion that ASD and psychosis may share underlying cognitive and neurobiological vulnerabilities, manifesting as different clinical syndromes across a spectrum.

In this context, the concept of the “broader autism phenotype” (BAP) has emerged, referring to subclinical autistic traits (ATs) observable in first-degree relatives of individuals with ASD. The BAP refers to the presence of subclinical traits of autism such as subtle communication difficulties, cognitive rigidity, and mild impairments in social reciprocity, initially studied in relatives of people with autism and later considered to be distributed in a continuum from the general to the clinical population [17]. These individuals typically maintain overall functional independence, making the identification of BAP challenging, particularly due to the intrinsic dimensional nature of the condition. Nonetheless, BAP has been associated with increased vulnerability to psychiatric disorders, including anxiety, mood, and obsessive–compulsive disorders, as well as with increased suicidality risk [17,18,19,20,21,22,23].

Taken together, these perspectives support the relevance of studying psychotic spectrum symptoms across the autism continuum, including both individuals with ASD and BAP. Investigating psychotic symptoms in these populations may clarify whether such features reflect true comorbid conditions, dimensional expressions of a shared vulnerability, or epiphenomena arising from overlapping cognitive and neurobiological mechanisms. Despite this growing interest, few studies have systematically compared psychotic spectrum features across individuals with different gradients of the autism spectrum. Even fewer have examined these associations using dimensional, spectrum-based psychometric tools that capture subthreshold symptoms across both domains.

The aim of the present study was to evaluate the presence and correlates of psychotic spectrum symptoms in individuals with ASD, their first-degree relatives (BAP), and healthy controls (HCs). Specifically, we sought to compare the severity and distribution of psychotic spectrum symptoms across the three groups, to assess the relationship between psychotic and ATs, and to explore whether psychotic spectrum symptom severity and dimensions were associated with diagnostic group membership in an exploratory, probabilistic framework.

## 2. Materials and Methods

### 2.1. Study Sample and Procedures

The study involved a sample of 22 adult patients diagnosed with ASD, recruited from both inpatient and outpatient settings at the Psychiatric Department of the Azienda Ospedaliera Universitaria Pisana, along with 22 first-degree relatives of ASD patients (BAP) and 24 HCs. Inclusion criteria for ASD group were as follows: age between 18 and 65, minimal to no intellectual or language developmental issues. For each patient, a parent or adult sibling was invited to join, forming the BAP group. First-degree relatives were included in the BAP group based on familial relationship independently from the presence of ASD symptoms. However, in a previous study, which examined a slightly wider portion of the same population used for the present work, the relatives’ group indeed reported higher ATs than HC [24]. Key exclusion criteria for both groups included diagnoses of schizophrenia, substance use disorder, neurodegenerative diseases, or any other significant medical or neurological condition, as well as any difficulty completing the examinations due to language or intellectual challenges. Additionally, BAP participants under 18 years or those with a DSM-5 diagnosis of ASD or any other neurodevelopmental disorder were excluded from the study. The HC group was recruited voluntarily, with the same exclusion criteria as the ASD group, plus the exclusion of anyone with mental disorder according to DSM-5 criteria. Recruitment and assessment procedures followed a standardized protocol across groups, including eligibility screening, informed consent, and supervised administration of questionnaires.

Before participation, all individuals were fully informed about the study and had the opportunity to ask questions before providing their signed consent. The study adhered to the Helsinki Declaration, and all procedures were approved by the local ethics committee.

Performing a power analysis, our sample power of 1.000 on the Psychotic Spectrum-Self Report (PSY-SR) total score and the observed power for PSY-SR domains and subdomains are reported in Table 1.

### 2.2. Measures

#### 2.2.1. The PSY-SR

The PSY-SR is a self-report tool designed to assess a broad range of symptoms associated with the psychotic spectrum, ranging from mild symptoms and subthreshold conditions to full-blown major psychosis. The instrument consists of 164 dichotomous items, divided into five domains: paranoid, including mild hypervigilance, distrust, suspiciousness, interpretive thinking, and paranoid self-reference; schizoid, covering religious beliefs, superstition, and unusual or magical thoughts; interpersonal sensitivity, describing the tendency to withdraw from others due to fear of misunderstanding or criticism; misperceptions, examining dissociative phenomena and borderline experiences of hallucinations and delusions; and typical symptoms. Within each domain, more specific subdomains are explored, including *hypertrophic* *self-esteem, strict thinking, superstition, fanaticism,* *relations with others, self-reference, interpretative attitude, suspiciousness, anger/over reactivity, hypervigilance, schizoidism–autism, schizotypy, illusions, depersonalization/derealization, delusions, hallucinations* and *catatonia* [25].

The PSY-SR was originally developed to assess psychotic symptoms dimensionally across a continuum ranging from subthreshold manifestations to full-blown psychotic disorders, rather than to provide categorical diagnoses. For this reason, the PSY-SR is not strictly tied to a specific DSM edition, and its core symptom domains (e.g., paranoid ideation, schizoid traits, perceptual disturbances, and typical psychotic symptoms) largely overlap across DSM-IV and DSM-5 conceptualizations of the psychotic spectrum. Previous validation studies have demonstrated good internal consistency (Kuder–Richardson coefficient >0.50 for all the domains and >0.70 for 12 out of 16 domains) and reliability of the instrument in adult clinical and nonclinical populations, including samples characterized by subthreshold or spectrum psychopathology. In the present study, the PSY-SR was therefore considered suitable for assessing dimensional psychotic spectrum features in adults with ASD, their first-degree relatives, and healthy controls.

#### 2.2.2. The AdAS Spectrum

The AdAS Spectrum is a self-report instrument made of dichotomous 160 items organized into seven domains, that investigates a broad range of autism-related symptoms and traits in individuals without cognitive or language impairments. Its domains address: *childhood and adolescence, verbal* and *non-verbal communication, empathy, rigidity and adherence to routine, restricted interests and rumination,* and *hyper- or hypo-reactivity to sensory stimuli*. The validation study showed strong internal consistency, excellent test–retest reliability, and convergent validity with other dimensional autism spectrum assessments [26].

### 2.3. Statistical Analysis

Group differences in socio-demographic variables were examined using statistical tests appropriate for the type and distribution of the data.

One-way analysis of variance (ANOVA) was used for comparison of normally distributed continuous variables such as age, while Chi-square tests were used to compare categorical variables like gender across groups.

A Kruskal–Wallis test was applied when normality assumptions were not met, specifically among results of psychometric measures. Kruskal–Wallis tests, followed by Bonferroni post hoc tests, were performed to compare AdAS Spectrum total scores and PSY-SR total and domain score across groups.

Afterwards, Spearman’s correlation analysis was performed in order to investigate the pattern of correlations between PSY-SR and AdAS Spectrum domains and total scores.

We then performed a multinomial logistic regression using the inclusion in the different diagnostic groups as the dependent variable and PSY-SR total scores as the independent variable in the first regression, in order to investigate whether the presence of psychotic symptoms was statistically predictive of the inclusion in ASD or BAP group with respect to HCs. An additional exploratory multinomial logistic regression was performed using the same dependent variable and PSY-SR domain scores as independent variables.

All statistical analyses were performed with SPSS version 26.0.

## 3. Results

The sample showed a significant difference between groups by sex (Chi-square = 16.06, *p* < 0.001), with males being more represented in the ASD group (M = 16, 72.7%; F = 6, 27.3%) and females in the BAP group (M = 3, 13.6%; F = 19, 86.4%), while the HC group showed a more balanced male-to-female ratio (M = 9, 37.5%; F = 15, 62.5%). Moreover, a significant age difference emerged between groups (F = 59.85, *p* < 0.001), with the BAP group (mean age = 55.14 ± 10.63) being significantly older than both the ASD (mean age = 28.14 ± 7.17) and HC groups (mean age = 33.29 ± 8.05), which did not significantly differ from each other in age.

Groups also significantly differed in terms of years of instruction (F = 23.198; *p* ≤ 0.001) with HCs reporting significantly more years of instruction (19.67 ± 3.00 years) compared to ASD (13.73 ± 2.43 years) and BAP (13.41 ± 4.78 years).

In addition, the Kruskal–Wallis test comparing total AdAS Spectrum scores confirmed the presence of significant differences in autistic spectrum symptoms across groups (H = 34.820, *p* < 0.001). Specifically, as expected, participants in the ASD group reported significantly higher scores (80.23 ± 22.39) than the other two groups, while the BAP group also showed significantly higher scores compared to the HCs (39.21 ± 14.16 vs. 20.83 ± 13.41). This pattern confirms the characterization of the relatives’ group as individuals with an intermediate autistic spectrum load, consistent with the conceptualization of the BAP.

**Figure 1:**
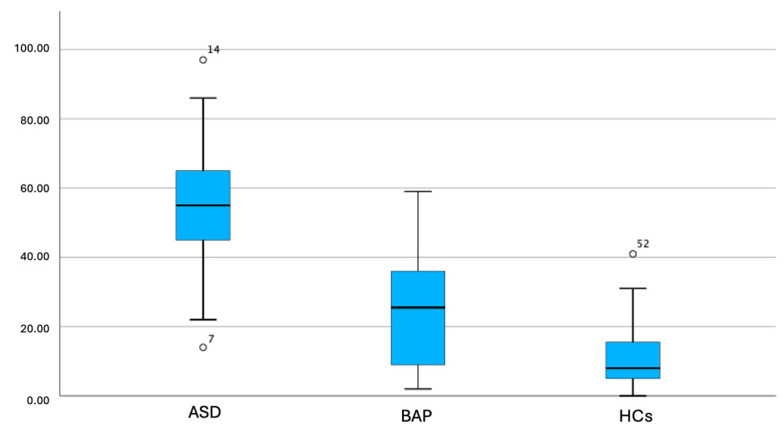
Box plot comparison of PSY-SR total scores among groups.

**Figure 2:**
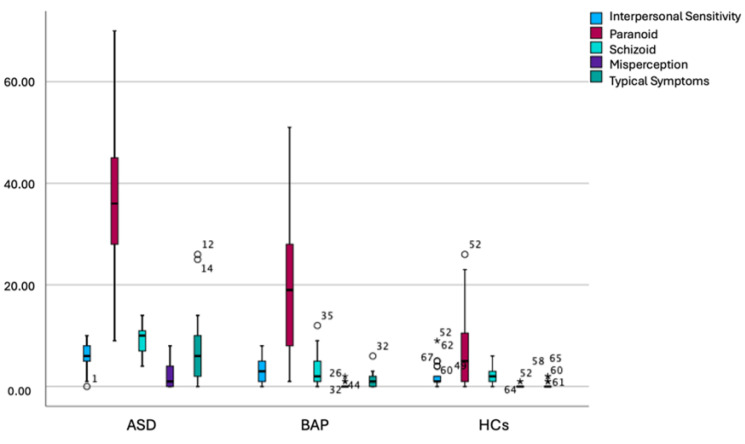
Box plot comparison of PSY-SR domains scores among groups.

Results from the Kruskal–Wallis analysis reported in Table 2 and box plow comparison reported in Figure 1 and Figure 2 showed that ASD participants reported significantly higher PSY-SR total, Paranoid domain subdomain score compared to BAP, which in turn scored significantly higher than HCs. Moreover, ASD participants reported significantly higher scores than BAP and HCs in the PSY-SR Interpersonal sensitivity domain, as well as in Strict thinking, Superstition, Relations with others, Self-reference, Suspiciousness, and Anger/over reactivity subdomains of Paranoid domain in the Schizoid domain as well as in all its subsections (Schizoidism-autism and Schizotypy) and in the Misperception domain and in all its subdomains (Illusions and Depersonalization/derealization) and in the Typical symptoms domain as well as in all its subdomains (Delusions, Hallucinations and Catatonia). BAP and HCs in turn did not significantly differ from one another. Similarly, ASD and BAP scored similarly and significantly higher than HCs, in the Hypertrophic self-esteem and Hypervigilance subdomain score. Lastly, ASD individuals scored significantly higher than HCs in the Fanaticism, Interpretative attitude and Hallucinations subsection; however, no significant differences were highlighted between BAP and ASD and BAP and HCs. All significant differences were maintained when applying Bonferroni post hoc correction with the only exception of PSY-SR Fanaticism.

Results from Spearman’s correlation analysis, highlighted that all PSY-SR domains were significantly positive correlated with all AdAS Spectrum domains (see Table 3).

In order to evaluate the specific association of psychotic symptoms as measured by PSY-SR total score with the two diagnostic groups (ASD and BAP), a multinomial logistic regression analysis was performed. Results showed that, when considering group category as dependent variable and in particular HCs as the reference category, a higher PSY-SR total score is significantly predictive of the inclusion in the ASD and BAP groups (see Table 4). Results from a second exploratory multinomial logistic regression analysis with PSY-SR domain scores as independent variables highlighted that considering group category as dependent variable and in particular HCs as the reference category, a higher PSY-SR Paranoid domain score was a significant predictor of the inclusion in the ASD and BAP groups (see Table 5).

## 4. Discussion

The aim of the present study was to assess presence and correlates of psychotic symptoms in individuals with ASD and their first-degree relatives.

Our findings revealed that individuals with ASD scored significantly higher on all PSY-SR domains and the total score compared to HCs. Similarly, the BAP group also showed significantly higher scores than HCs on most PSY-SR domains, suggesting that also first-degree relatives of ASD probands, a group typically characterized by a higher presence of ATs, are more likely to experience psychotic symptoms. Although systematic research on the co-occurrence of ASD and psychosis is still lacking, our results are in line with some studies reporting elevated rates of co-occurrence [27,28,29]. Indeed, recent studies reported an estimated prevalence of schizophrenia in ASD subjects ranging from 0% to 6% [30], as well as evidence that psychotic experiences are two to five times more prevalent among individuals with ASD or elevated ATs compared to the general population, with delusions occurring at a significantly higher rate in individuals with ASD, whereas the prevalence of hallucinations appeared to be comparable between groups [13]. Some studies also highlighted how some central features of ASD, such as atypical sensory experiences, can resemble psychotic manifestations. For example, in terms of alterations in sensor perception, the diagnosis of autism itself includes the presence of unusual patterns of perceptions that are often difficult to distinguish from real hallucinations. In particular, auditory dysperceptive phenomena, where individuals with ASD may perceive sounds, like the arrival of a train, well before they actually occur, can potentially resemble auditory hallucinations [31,32]. Moreover, the difficulty in describing sensory processing is compounded by verbal communication deficits, which hinder proper reporting of these experiences [32,33]. Additionally, negative symptoms such as alogia, emotional flattening, and catatonic behavior are common to both ASD and psychosis [30]. As for BAP, it has also been linked in the literature to the phenomenon of psychosis, presenting a higher prevalence than the general population and confirming the link between the psychotic and autistic spectra, even in its more nuanced manifestations in which aspects of cognitive rigidity or difficulties in the social sphere prevail, without language impairment [30].

Noticeably, while all psychotic spectrum symptoms, including typical symptoms and misperceptions, were higher in ASD participants with respect to HCs, global paranoid symptoms and some symptoms related to interpersonal sensitivity such as hypertrophic self-esteem, interpretative attitude and hypervigilance were significantly higher with respect to HCs also in the BAP group: among them, interpretative attitude and hypertrophic self-esteem were on a similar level in both BAP and ASD. The specific presence of the symptoms investigated in these domains within the BAP has not been thoroughly examined in the literature, thus representing an area deserving of further investigation [32]. If their presence is detectable through dimensional instruments such as the PSY-SR, it is plausible that, similarly to other psychopathological traits that have already been extensively studied, these symptoms may also contribute to the cognitive and social difficulties that characterize the BAP. These findings are consistent with previous literature, which has highlighted that the presence of affective disorders and schizophrenia in parents increases the risk of ASD in their offspring [34,35]. Interestingly, a similar pattern is observed in siblings: having a sibling with schizophrenia or ASD also elevates the risk of developing ASD [35,36,37]. Many other studies have further supported this, showing that the presence of schizophrenia and bipolar disorder in siblings is a risk factor for the development of ASD [35,38], as well as reporting a correlation between schizophrenia-like psychosis in parents and the subsequent development of ASD in their children [39]. On the other hand, research has also demonstrated that parents of children with ASD face a higher risk of hospitalization for severe psychiatric conditions, with a notably higher risk for schizophrenia compared to healthy controls [40]. According to various authors, these findings suggest the presence of a shared genetic substrate within the family unit (parents, children, and siblings) or an interaction between genetic predispositions and environmental factors that may contribute to the emergence of these disorders [38,41,42].

The intermediate psychotic spectrum symptom levels observed in the BAP group should be interpreted with caution. Because BAP participants were first-degree relatives of individuals with ASD, their profiles may reflect a combination of shared genetic liability and familial environmental influences, rather than solely the expression of subclinical ATs. Shared neurodevelopmental vulnerabilities, family-level stressors, or learned cognitive–emotional styles may all contribute to the observed pattern.

These findings were supported by Pearson correlation analysis, which demonstrated that all PSY-SR domains and the total score were positively correlated with all AdAS Spectrum domains and the total score. The observed gradient across ASD, BAP, and HC groups suggests that subthreshold psychotic features may represent part of a broader neurodevelopmental vulnerability rather than discrete comorbidity. One possible explanation for these findings lies in shared neurodevelopmental mechanisms linking ATs and psychotic spectrum symptoms, including atypical social cognition, altered perceptual processing, and heightened threat attribution. These overlapping features may contribute to both the interpersonal difficulties characteristic of ASD and the emergence of subthreshold paranoid ideation or unusual perceptual experiences [14,32]. Interestingly, the domain that exhibited the strongest correlations was PSY-SR Schizoid domain, in line with the existing literature, which highlights a strong association between autism spectrum and schizoid and schizotypal traits [43,44]. Notably, a 2011 study demonstrated that schizotypal traits were more pronounced in individuals with ASD compared to healthy controls, manifesting not only as negative symptoms but also positive symptoms and disorganized speech and behavior [45]. Similarly, a 2020 study focusing on the schizoid dimension found that while only a small proportion of individuals with ASD met the criteria for schizoid personality disorder, schizoid traits were significantly more prominent in those with ASD than in healthy controls [46]. On the other hand, interestingly, the only domains that did not show correlations were the PSY-SR Typical Symptoms domain and the AdAS Spectrum Empathy domain. This finding seems to contradict the existing literature, which has reported deficits in both cognitive and affective empathy across the psychotic spectrum [47,48,49,50]. However, the relationship between these factors remains unclear, and there is ongoing debate as to whether the empathy deficits observed can be attributed to symptoms of anxiety and depression, which often co-occur with psychosis [51,52]. One possible explanation for this lack of correlation may lie in the fact that empathy deficit and alterations may sometimes be considered as part of the negative symptoms of psychosis, whereas, PSY-SR Typical Symptoms domain focuses specifically on positive symptoms, such as delusions and hallucinations, which may not correlate with the negative ones.

Lastly, results from the exploratory regression analyses showed that overall psychotic symptoms as measured by PSY-SR domain scores were statistically predictive of belonging to ASD or BAP groups. These findings align with previous longitudinal studies that reported 25% of children diagnosed with childhood-onset schizophrenia or psychosis later received an ASD diagnosis during their lifetime [53]. Subsequent research confirmed these results, with a similar percentage (28%) of individuals also receiving an ASD diagnosis [54]. Furthermore, a study by Hallerbäck and colleagues [55] involving a Swedish cohort of patients with schizophreniform illness found that 41% met the diagnostic criteria for ASD, as determined through parent interviews, with a focus on ASD symptoms observed during childhood and adolescence. These findings suggest that the presence of autism or significant ATs may serve as a risk factor for the later development of either full-blown or milder psychotic symptoms. Moreover, considering that psychotic symptoms also predicted the inclusion in the BAP group, our data seem to support the association of psychotic symptoms even with subthreshold autism spectrum manifestations, which thus may be regarded as a condition with relevant psychopathological correlates [15,16,27].

Finally, the evidence that a higher Paranoid score was a predictor for inclusion in the ASD and BAP groups reinforces the connection between the paranoid dimension—which encompasses traits like rigid thinking, fanaticism, anger, and hypersensitivity to stimuli—and autism spectrum [56,57]. Indeed, individuals with paranoid traits may have inflexible beliefs, particularly around distrust or suspicion of others and similarly, individuals with autism often exhibit cognitive rigidity, characterized by a preference for routines, difficulties with flexibility in thinking, and challenges in adapting to change. Similarly, people with paranoid traits often have heightened sensitivity to perceived threats in their social environment and individuals with autism may struggle with social communication and interpreting social cues, sometimes leading to misunderstandings or misinterpretations of social situations. Overall, the presence of cognitive rigidity, along with heightened sensitivity to external stimuli and difficulties in emotional processing, are central features of the autistic core, as outlined in the diagnostic criteria for ASD, may be correlated to the development of paranoidal symptomatology. Therefore, a more pronounced expression of these traits is indicative of a higher likelihood of exhibiting ATs, whether subtle or more pronounced [58].

These findings should be considered in the context of several limitations. First, the diagnostic groups differed significantly in age and sex distribution, with the ASD group being younger and predominantly male and the BAP group being older and predominantly female. As age and sex have been shown to influence both the expression and reporting of ATs and psychotic-like experiences, demographic factors may have partially contributed to the observed group differences in PSY-SR scores. Moreover, the study’s cross-sectional design limits our ability to draw conclusions about cause-and-effect relationships or the temporal progression with respect to the investigated variables. Longitudinal studies will be essential to clarify developmental trajectories and potential causal pathways. Additionally, the exclusive reliance on self-report measures may have introduced response bias, particularly among individuals with ASD, who may encounter difficulties in interpreting items or reporting subjective internal experiences. Future research would benefit from a multi-method assessment approach. Furthermore, the small sample size reduces the ability to generalize the results, making it challenging to apply these findings to larger or more diverse populations. Larger, demographically balanced samples will be necessary to confirm the present findings and to permit more refined modeling strategies. An additional limitation concerns the potential non-independence of observations, as individuals in the BAP group were first-degree relatives of participants in the ASD group. Although this design may introduce some degree of familial clustering, each ASD participant contributed at most one relative, resulting in dyadic associations rather than hierarchical clustering within diagnostic groups. The main analyses focused on between-group comparisons and dimensional associations across the total sample, rather than on within-family effects. Given the small sample size, the application of cluster-informed or multilevel models was not feasible without a substantial loss of statistical power and model stability. Nevertheless, future studies with larger family-based samples should explicitly account for familial clustering to better disentangle shared genetic or environmental influences. Additionally, correlations between psychotic spectrum symptoms and ATs were computed across the full sample to capture dimensional associations across the autism spectrum; however, between-group differences may have contributed to the observed relationships. Larger studies will be needed to examine these associations within diagnostic groups and using covariate-adjusted models. Lastly, pharmacological treatments, particularly antipsychotic medications, and the presence of a comorbid psychiatric disorder, may influence the expression and reporting of psychotic spectrum symptoms, but, even in this case, the limited sample size prevented us from performing therapy and comorbidity-informed models. Future studies should include stratifications for comorbidity and pharmacotherapy to clarify their potential impact.

## 5. Conclusions

This study contributes to the growing body of evidence linking autism and psychosis spectra, which have historically been studied as similar entities. Over time, these conditions have been recognized as distinct but deeply interconnected, likely due to shared genetic, epigenetic, and environmental factors. In addition to reinforcing this connection, the study highlights the relationship between the BAP and psychosis, suggesting that subthreshold autistic traits in relatives of individuals with ASD may be associated with psychotic symptoms. This expands the scope of current literature, offering a broader perspective on the familial and genetic links between these conditions.

The manifestations of the autism spectrum can overlap with those of the psychotic spectrum, potentially spanning generations with varying degrees of intensity. Further research is needed to clarify the relationship between these two domains, particularly to facilitate accurate recognition and possibly earlier diagnosis, both in individuals with ASD and their family members. Additionally, a deeper understanding of the clinical complexities involved will be crucial for providing appropriate treatment, taking into account the neurodevelopmental basis that transcends generations.

## Figures and Tables

**Table 1:** Observed power for PSY-SR domains and subdomains.

PSY-SR Domains	Observed Power	PSY-SR Subdomains	Observed Power
Interpersonal sensitivity	1.000		
Paranoid	1.000	Hypertrophic self-esteem	0.999
		Strict thinking	1.000
		Superstition	0.970
		Fanaticism	0.892
		Relations with others	0.999
		Self-reference	1.000
		Interpretative attitude	0.987
		Suspiciousness	0.999
		Anger/over reactivity	1.000
		Hypervigilance	1.000
Schizoid	1.000	Schizoidism–autism	0.999
		Schizotypy	1.000
Misperceptions	0.999	Illusions	0.993
		Depersonalization /derealization	0.999
Typical symptoms	1.000	Delusions	1.000
		Hallucinations	0.909
		Catatonia	0.972

**Table 2:** Comparison of PSY-SR scores among ASD, BAP and HCs.

	ASD Mean ± SD, Mean Rank	BAP Mean ± S, Mean Rank	HCs Mean ± S, Mean Rank	H	*p*	Post Hoc Bonferroni *p*	η^2^	CI (95%)	
								Lower Bound	Upper Bound
***INTERP*** ***. SENSITIV. tot. score***	6.00 ± 2.63, 50.26	2.91 ± 2.11, 30.86	1.83 ± 2.14, 22.65	23.936	<0.001 *	<0.001 *	0.379	0.184	0.513
*Hypertrophic self-esteem*	3.86 ± 2.13, 46.90	2.73 ± 1.98, 37.75	0.87 ± 1.07, 19.27	24.411	<0.001 °	<0.001 °	0.340	0.148	0.479
*Strict thinking*	4.38 ± 2.01, 49.90	2.23 ± 2.04, 32.20	1.08 ± 1.25, 21.73	24.594	<0.001 *	<0.001 *	0.377	0.183	0.512
*Superstition*	1.62 ± 1.56, 45.21	0.45 ± 0.59, 29.39	0.46 ± 0.72, 28.42	12.131	0.002 *	0.046 *	0.222	0.055	0.369
*Fanaticism*	2.86 ± 2.59, 41.79	1.86 ± 1.67, 35.84	0.92 ± 0.83, 25.50	8.580	0.014 ^^^	0.322	0.168	0.024	0.313
*Relations with others*	3.71 ± 2.15, 48.14	1.91 ± 2.20, 32.66	0.65 ± 0.88, 20.93	23.208	<0.001 *	<0.001 *	0.329	0.136	0.470
*Self-reference*	2.95 ± 1.46, 51.29	1.14 ± 1.46, 31.16	0.37 ± 0.71, 21.48	29.501	<0.001 *	<0.001 *	0.438	0.243	0.563
*Interpretative attitude*	3.86 ± 2.59, 45.86	2.45 ± 2.46, 35.41	0.83 ± 1.37, 22.33	17.398	<0.001 ^^^	<0.001 ^^^	0.252	0.077	0.399
*Suspiciousness*	6.81 ± 4.37, 49.10	3.41 ± 3.79, 33.57	0.96 ± 1.73, 21.19	24.096	<0.001 *	<0.001 *	0.338	0.146	0.477
*Anger/over react.*	4.28 ± 2.00, 53.64	1.27 ± 1.55, 29.16	0.50 ± 0.93, 21.25	35.806	<0.001 *	<0.001 *	0.538	0.355	0.644
*Hypervig.*	4.14 ± 2.94, 48.62	1.91 ± 1.66, 35.45	0.46 ± 0.78, 19.88	26.275	<0.001 °	<0.001 °	0.385	0.190	0.518
***PARANOID total score***	38.48 ± 15.13, 51.95	19.36 ± 12.8, 33.32	7.13 ± 7.45, 16.83	36.810	<0.001 ^§^	<0.001 ^§^	0.541	0.356	0.647
*Schizoidism-autism*	5.38 ± 2.25, 50.45	2.68 ± 2.82, 28.02	1.92 ± 1.35, 25.08	22.674	<0.001 *	<0.001 *	0.319	0.130	0.460
*Schizotypy*	3.76 ± 2.36, 52.14	0.82 ± 1.26, 28.89	0.25 ± 0.53, 22.81	32.380	<0.001 *	<0.001 *	0.506	0.318	0.619
***SCHIZOID total score***	9.14 ± 2.95, 53.86	3.50 ± 3.46, 27.75	2.17 ± 1.68, 22.35	33.153	<0.001 *	<0.001 *	0.550	0.369	0.654
*Illusions*	1.53 ± 1.86, 46.38	0.18 ± 0.50, 29.32	0.08 ± 0.28, 27.46	20.568	<0.001 *	<0.001 *	0.269	0.089	0.414
*Depers./dereal.*	0.62 ± 0.74, 44.95	0.00 ± 0.00, 29.00	0.00 ± 0.00, 29.00	22.225	<0.001 *	<0.001 *	0.335	0.144	0.475
***MISPERCEPTIONS total score***	2.14 ± 2.43, 47.74	0.18 ± 0.50, 28.66	0.08 ± 0.28, 26.88	24.293	<0.001 *	<0.001 *	0.318	0.128	0.495
*Delusions*	5.47 ± 5.51, 50.14	1.00 ± 1.48, 31.98	0.21 ± 0.51, 21.73	28.024	<0.001 *	<0.001 *	0.348	0.155	0.486
*Hallucinations*	1.43 ± 2.38, 40.98	0.14 ± 0.35, 31.66	0.08 ± 0.28, 30.04	8.414	0.015 ^^^	0.345	0.176	0.028	0.322
*Catatonia*	0.43 ± 0.68, 41.67	0.00 ± 0.00, 30.50	0.00 ± 0.00, 30.50	16.825	<0.001 *	<0.001 *	0.225	0.057	0.372
***TYPICAL SYMPTOMS total score***	7.33 ± 7.38, 50.83	1.14 ± 1.46, 31.93	0.29 ± 0.62, 21.17	29.430	<0.001 *	<0.001 *	0.360	0.166	0.479
**PSY-SR total score**	55.76 ± 20.09, 52.83	25.95 ± 16.8, 31.50	11.26 ± 10.1, 17.76	37.036	<0.001 ^§^	<0.001 ^§^	0.579	0.402	0.677

* ASD > BAP, HCs; ° ASD, BAP > HCs; ^^^ ASD > HCs; ^§^ ASD > BAP > HCs; significant for *p* < 0.05.

**Table 3:** Spearman’s correlations coefficients heat map among PSY-SR domains and total score and AdAS Spectrum domains and total score in the total sample.

	Child./ Adolesc.	Verb. Comm.	Non-Verb. Comm.	Emp.	Inflex. and Routine	Restrict. Int. and Rum.	React.	AdAS Spec. Tot. Score
**Int. sens.**	0.343 **	0.433 **	0.361 **	0.313 *	0.441 **	0.444 **	0.376 **	0.466 **
**Paranoid**	0.381 **	0.483 **	0.472 **	0.403 **	0.520 **	0.427 **	0.421 **	0.554 **
**Schizoid**	0.412 **	0.511 **	0.532 **	0.438 **	0.522 **	0.474 **	0.517 **	0.573 **
**Mispercept.**	0.359 **	0.440 **	0.390 **	0.391 **	0.356 **	0.336 *	0.430 **	0.443 **
**Typ. sympt.**	0.333 *	0.497 **	0.519 **	0.351 **	0.495 **	0.421 **	0.416 *	0.533 **
**PSY-SR tot. score**	0.409 **	0.514 **	0.500 **	0.411 **	0.539 **	0.457 **	0.450 **	0.576 **

* Significant for *p* < 0.05; ** significant for *p* < 0.01.

**Table 4:** Multinomial logistic regression analysis with group category as the dependent variable and PSY-SR total scores as independent variable.

		B (S.E.)	EXP (B)	CI (95%)		*p*
				Lower Limit	Upper Limit	
**ASD**	*Intercept*	−5.148 (1.256)				<0.001 *
	**PSY-SR total score**	0.164 (0.037)	1.179	1.096	1.268	<0.001 *
**BAP**	*Intercept*	−1.423 (0.558)				0.011 *
	**PSY-SR total score**	0.078 (0.028)	1.081	1.024	1.141	0.005 *

Cox and Snell R^2^: 0.559; Nagelkerke R^2^: 0.630; McFadden R^2^: 0.360; * Significant for *p* < 0.05.

**Table 5:** Multinomial logistic regression analysis with group category as the dependent variable and PSY-SR domains scores as independent variables.

		B (S.E.)	EXP (B)	CI (95%)		*p*
				Lower Limit	Upper Limit	
**ASD**	*Intercept*	−4.578 (1.368)				0.001 *
	**Interpersonal sensitivity**	−0.192 (0.318)	0.825	0.442	1.538	0.545
	**Paranoid**	0.220 (0.109)	1.246	1.006	1.544	0.044 *
	**Schizoid**	0.086 (0.235)	1.090	0.688	1.726	0.713
	**Misperceptions**	0.159 (0.607)	1.173	0.357	3.853	0.793
	**Typical symptoms**	0.249 (0.509)	1.283	0.474	3.477	0.624
**BAP**	*Intercept*	−1.423 (0.620)				0.022 *
	**Interpersonal sensitivity**	−0.461 (0.272)	0.631	0.370	1.075	0.090
	**Paranoid**	0.257 (0.093)	1.293	1.076	1.552	0.006 *
	**Schizoid**	−0.229 (0.203)	0.795	0.534	1.184	0.259
	**Misperceptions**	−0.495 (0.812)	0.610	0.124	2.995	0.542
	**Typical symptoms**	0.072 (0.486)	1.074	0.414	2.786	0.883

Cox and Snell R^2^: 0.628; Nagelkerke R^2^: 0.707; McFadden R^2^: 0.446; *: Significant for *p* < 0.05.

## Data Availability

The datasets generated during and/or analyzed during the current study are available from the corresponding author upon reasonable request due to privacy and ethical restrictions.

## Abbreviations

The following abbreviations are used in this manuscript:

ASD	Autism Spectrum Disorder
DSM	Diagnostic and Statistical Manual for Mental Disorder
BAP	Broad Autism Phenotype
HCs	Healthy controls
PSY-SR	Psychotic Spectrum-Self Report
AdAS Spectrum	Adult Autism Subthreshold Spectrum
ToM	Theory of Mind
